# A Rare Presentation of Gastrointestinal Stromal Tumor Causing Gastroduodenal Intussusception

**DOI:** 10.7759/cureus.34632

**Published:** 2023-02-04

**Authors:** Ariana R Tagliaferri, Minha Naseer, Gabriel Melki, Shoaib Azam, Yana Cavanagh

**Affiliations:** 1 Internal Medicine, St. Joseph’s Regional Medical Center, Paterson, USA; 2 Internal Medicine, St. Joseph’s University Medical Center, Paterson, USA; 3 Medicine, St. Joseph’s University Medical Center, Paterson, USA; 4 Gastroenterology and Hepatology, St. Joseph’s University Medical Center, Paterson, USA; 5 Gastroenterology, St. Joseph’s Regional Medical Center, Paterson, USA

**Keywords:** gist, malignancy, gastroenterology, endoscopic repair, surgery, intussusception, gastroduodenal

## Abstract

Adult intussusception is exceedingly rare and most commonly occurs in the stomach or ileum. It is less common for adult intussusception to be classified as gastroduodenal, which also carries a higher mortality rate. Adult intussusception usually warrants surgical intervention as the underlying cause is often malignancy. However, rarely, the etiology is a gastrointestinal stromal tumor (GIST). Here, we present the case of a patient who presented with abdominal pain, vomiting, and hemorrhagic shock and was diagnosed with gastroduodenal intussusception secondary to a gastric GIST.

## Introduction

Although intussusception is a common diagnosis in children, it is rare in adults with only 2-3/100,000 cases diagnosed annually [1,2]. Child intussusception is usually idiopathic and occurs in children aged 4-10 months [2]. Childhood intussusception usually occurs in the ileum, but rarely involves the stomach, colon, or duodenum [2]. Conversely, adult intussusception is often caused by a definable structural lesion, is usually pathologic, and most commonly occurs in the stomach or small bowel [2,3]. Based on the anatomic location, intussusception can be classified as entero-enteric, in which it is confined to the small intestine, colic-colic, in which it is confined to the large intestine, ileo-colic, in which the ileum prolapses into the ascending colon, or ileo-cecal [2]. Intussusception is classified as gastroduodenal when the stomach invaginates through the pylorus and duodenum, but this is very rare and only occurs in up to 10% of all adult intussusception cases [2,3]. Thus, the gastroduodenal type is the least common type of adult intussusception. In general, adult intussusception of any type can be caused by Meckel’s diverticulum, colonic diverticulum, lipomas, Menetrier’s disease, strictures, polyps, or inflammatory lesions [1,3]. However, as stated above, the majority of cases are due to benign or malignant tumors, such as lymphomas, Brunner gland hamartomas, gastric carcinoma, or gastrointestinal stromal tumors (GISTs) [1,3,4].

GISTs are mesenchymal tumors that arise from the interstitial cells of Cajal [1]. Although they often occur in the small bowel and stomach, GISTs only account for 1% of gastroduodenal-type intussusceptions and are thus very rare [1,4]. Here, we report the case of an elderly patient who presented with vomiting and abdominal pain and was diagnosed with GIST after an episode of hemorrhagic shock, ultimately requiring surgical resection for intussusception.

## Case presentation

An 85-year-old male with hypercholesterolemia and cognitive impairment since childhood presented for non-bilious emesis and periumbilical abdominal pain lasting one day. Given the patient’s cognitive impairment, it is unknown whether he underwent routine preventive screenings during annual examinations, including colonoscopy or prostate evaluation. On arrival, he was afebrile and hemodynamically stable with examination only notable for diffuse abdominal tenderness without peritonitis.

Laboratory studies were significant for hypercalcemia, lactic acidosis, leukocytosis, and normocytic anemia (Table 1).

**Table 1:** Abnormal laboratory studies.

Laboratory abnormality	Laboratory value	Reference range
Calcium	10.9 mg/dL	8.6–10.3 mg/dL
Lactic acid	2.8 mmol/L	0.8–2.2 mmol/L
White blood cell count	14.0 × 10^3^/mm^3^	4.5–11 × 10^3^/mm^3^
Hemoglobin	11.7 g/dL	13.5–17.5 g/dL

**Figure 1:**
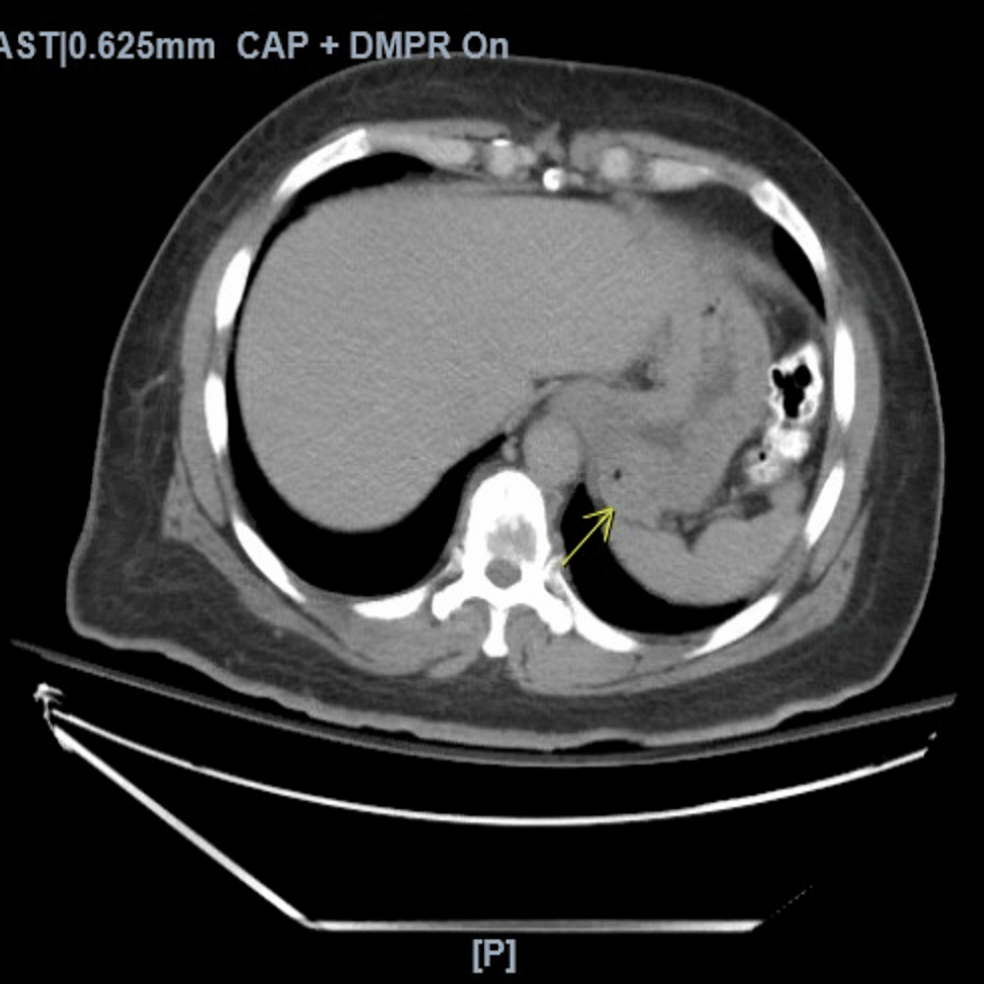
Axial computerized tomography of the abdomen and pelvis with intravenous and oral contrast. The stomach is opacified with oral contrast and there is a 5.6 × 5.3 cm mass arising from the body of the stomach, indicated by the arrow. The antrum wall is slightly thickened. There is no evidence of abdominal or pelvic lymphadenopathy.

Computerized tomography (CT) of the abdomen and pelvis without contrast revealed a heterogeneous non-obstructing mass within the stomach extending into the duodenum and an osteolytic lesion on T10 concerning for metastatic disease (Figure 1).

**Figure 2:**
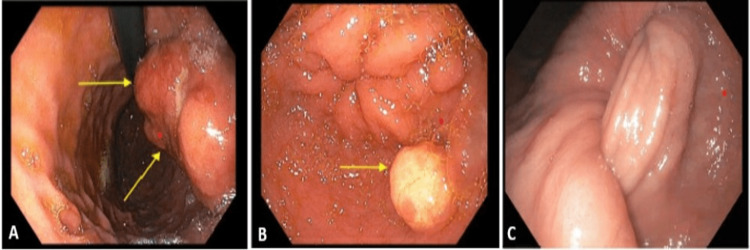
Esophagogastroduodenoscopy demonstrating gastroduodenal intussusception. (A) Large, fungating, pedunculated mass found in the gastric body, indicated by arrows. (B) Arrows indicate a single 15 mm nodule was found in the gastric antrum. (C) Gastroduodenal intussusception upon subsequent esophagogastroduodenoscopy.

He underwent an esophagogastroduodenoscopy (EGD) which showed a large, fungating, and ulcerated, partially circumferential mass involving two-thirds of the luminal gastric circumference (Figure 2).

**Figure 3:**
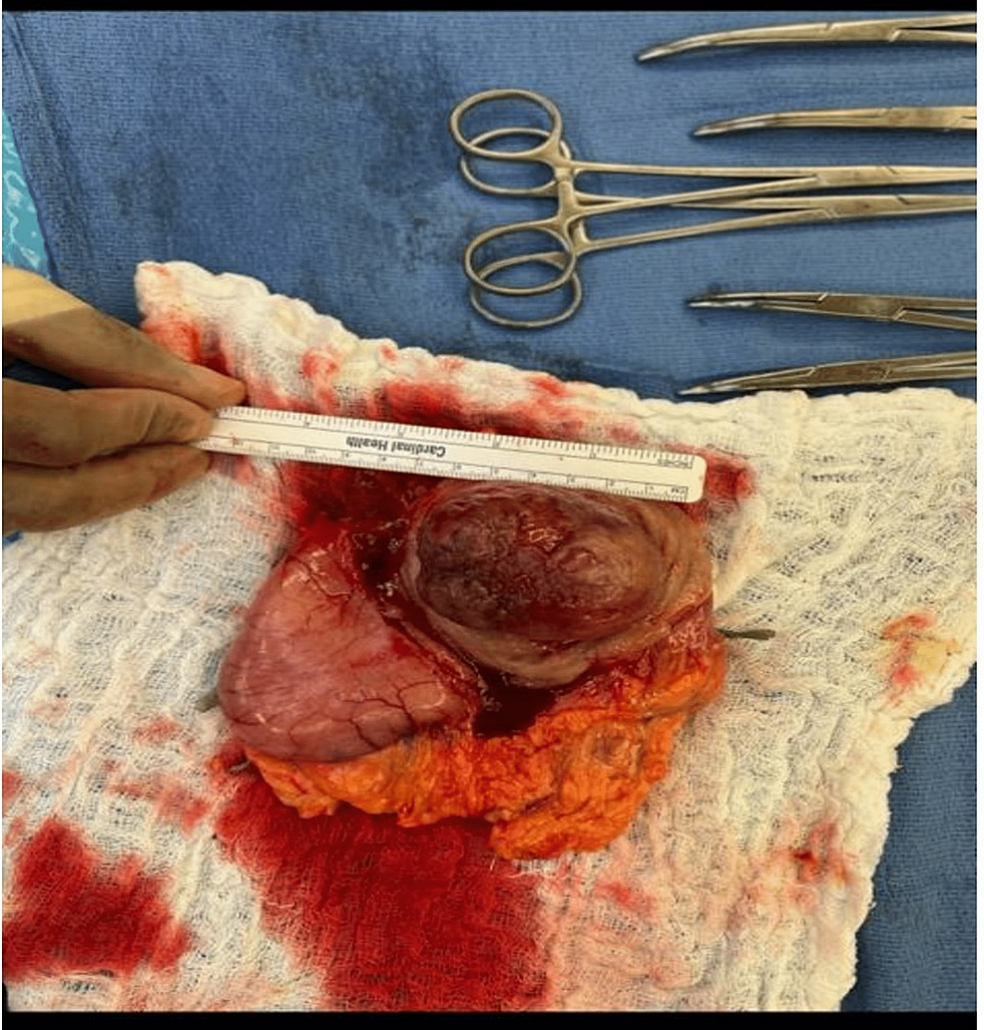
Resected submucosal tumor. Resected gastrointestinal stromal tumor measuring approximately 5.5 × 5.0 × 3.5 cm.

There were no stigmata of recent bleeding within the gastric body, but there was a single 15 mm papule in the gastric antrum. The stalk of the mass pushed past the pylorus into the duodenum, concerning for intussusception, and spontaneously reduced during endoscopic evaluation. The mass, however, was too large to fully resect. Biopsies of the mass and papule revealed hyperplastic changes and mildly chronic active antral gastritis with focal intestinal metaplasia, negative for *Helicobacter pylori*. Further imaging revealed osseous metastatic disease on nuclear medicine bone scan, which could not be biopsied due to neurovascular involvement. Given the spinal lesions and elevated prostate-specific antigen (PSA) of 64 µg/dL, he was evaluated by Urology. However, it was presumed that he had two primary malignancies because the PSA was not elevated enough for metastatic disease. During his hospitalization, the patient became encephalopathic and hypotensive from hemorrhagic shock after having multiple, voluminous bloody bowel movements. He was transfused blood products and colloid fluids and required pressor support in the Intensive Care Unit. To localize the bleeding, a CT angiogram of the abdomen and pelvis was performed and demonstrated gastroduodenal intussusception with active bleeding. He subsequently underwent an emergent exploratory laparotomy with partial gastrectomy, distal antrectomy, and Billroth II reconstruction (Figure 3).

Consent was obtained from his legal guardian since childhood. Final pathology results from the resected tumor revealed GIST, pathologically staged TP3N0. The spindle cells were positive histologically for CD117, CD34, and Desmin and negative for S100. The patient was initiated on imatinib therapy, given his bony metastasis.

## Discussion

GIST generally occurs in patients between the ages of 60 and 65 years old, and is very rare below the age of 40, unless there is concomitant intussusception, in which the mean age of diagnosis is approximately 33.6 years [4,5]. Irrespective of concomitant intussusception, generally, GISTs manifest in the ileum or jejunum in up to 30% of cases, 5% occur in the duodenum, 5% occur in the rectum, and less than 1% are located in the esophagus [5]. Only one study has reported an incidence of greater than 50% of intussusceptions caused by GISTs to be classified as gastroduodenal; however, these findings contradict previously published literature [1].

Our patient’s age was significantly higher than the mean age described in any aforementioned study, and our patient had a gastroduodenal intussusception [1,4]. With both intussusception and GIST, up to 83% of patients present with vomiting and abdominal pain, similar to our patient [1,2]. Although melena is also common, hemorrhagic shock is often not observed [6]. Only one other case report describes this, which makes our case unique [6]. Additionally, dyspepsia or ulcer-like symptoms are the most common manifestation of GIST alone due to pressure necrosis and mucosal ulceration; however, neither of these occurred in our patient [1,4]. Nonetheless, it is very rare for GIST to present with bowel obstruction or hemoperitoneum [3,5].

Previous studies described the mean size of the tumors causing intussusception to be 6.2 cm, which is larger than the GISTs found in the absence of intussusception (<5 cm) [1,3]. Our patient had a 5.5 × 5.0 × 3.5 cm submucosal mass surgically resected, with a pathology report confirming a low risk for tumor progression (3.6%). The National Institutes of Health and Fletcher’s Criteria are tools used for risk stratification and prognostication, given the spectrum of neoplastic and metastatic potential in GISTs [3,5]. The age of the patient at the time of diagnosis, tumor size <5 cm, and mitotic index <5 per 50 HPFs confer a good prognosis; however, the presence of tumor ulceration and necrosis and/or metastatic disease at the time of diagnosis carries a high mortality rate [1,5,7]. It was determined that our patient had two primary malignancies rather than metastatic involvement from the GISTs, thus preserving his calculated low-risk score.

The estimated five-year survival for patients with GIST alone is 61%; however, the survival rate for those with concomitant intussusception is unknown [7]. Though there was a low risk of progression identified pathologically in our patient, the location of his tumor and metastatic disease warranted adjuvant imatinib therapy, which historically has been used only in advanced disease [1].

## Conclusions

Our case of a GIST causing gastroduodenal intussusception is rare. Although the presentation with vomiting, abdominal pain, and melena is common in these patients, the subsequent hemorrhagic shock and age of our patient make our case unique, as it has only been described in one other case report. When GIST is the cause of intussusception, it does not often manifest as the gastroduodenal type. In patients with a well-circumscribed gastrointestinal mass in conjunction with intussusception, GIST should be entertained.
